# Leveraging the coronary calcium scan beyond the coronary calcium score

**DOI:** 10.1007/s00330-017-5264-3

**Published:** 2018-01-30

**Authors:** Daniel Bos, Maarten J. G. Leening

**Affiliations:** 1 Department of Radiology and Nuclear Medicine, Erasmus MC – University Medical Centre Rotterdam, Rotterdam, The Netherlands; 2 Department of Epidemiology, Erasmus MC – University Medical Centre Rotterdam, P.O. Box 2040, 3000 CA Rotterdam, The Netherlands; 3 Department of Epidemiology, Harvard T.H. Chan School of Public Health, Boston, MA USA; 4 Department of Cardiology, Erasmus MC – University Medical Centre Rotterdam, Rotterdam, The Netherlands

**Keywords:** Coronary artery calcium score, Atherosclerosis, X-ray computed tomography, Biomarkers, Preventive medicine

## Abstract

**Abstract:**

Non-contrast cardiac computed tomography in order to obtain the coronary artery calcium score has become an established diagnostic procedure in the clinical setting, and is commonly employed in clinical and population-based research. This state-of-the-art review paper highlights the potential gain in information that can be obtained from the non-contrast coronary calcium scans without any necessary modifications to the scan protocol. This includes markers of cardio-metabolic health, such as the amount of epicardial fat and liver fat, but also markers of general health including bone density and lung density. Finally, this paper addresses the importance of incidental findings and of radiation exposure accompanying imaging with non-contrast cardiac computed tomography. Despite the fact that coronary calcium scan protocols have been optimized for the visualization of coronary calcification in terms image quality and radiation exposure, it is important for radiologists, cardiologists and medical specialists in the field of preventive medicine to acknowledge that numerous additional markers of cardio-metabolic health and general health can be readily identified on a coronary calcium scan.

**Key Points:**

• *The coronary artery calcium score substantially increased the use of cardiac CT.*

• *Cardio-metabolic and general health markers may be derived without changes to the scan protocol.*

• *Those include epicardial fat, aortic valve calcifications, liver fat, bone density, and lung density.*

• *Clinicians must be aware of this potential additional yield from non-contrast cardiac CT.*

## Introduction

Over the past decade, non-contrast cardiac computed tomography (CT) has become an established diagnostic tool in clinical practice. The main purpose of these coronary calcium scans is to obtain the coronary artery calcium score (CACS) [1, 2], which is associated with a graded increased risk of future coronary events, heart failure and mortality [3–5], and even relates to dementia, cancer and kidney disease [6, 7]. On the other hand, a negative or zero CACS denotes a mid- to long-term risk of coronary events that is close to zero [8, 9]. As such, the current ACC/AHA guidelines on assessment of cardiovascular risk state that assessment of CACS may be considered based on a large number of observational studies: with a CACS of ≥ 300 Agatston units (or ≥ 75th percentile for age, sex and ethnicity) supporting an upward revision in risk assessment [10]. A range of alternative approaches to application of CACS for risk stratification in primary prevention has been proposed recently [11–13].

Most clinical radiologists and cardiologists will be aware of other cardiac imaging properties that can be obtained from coronary calcium scans, such as large myocardial scars or dimensions of the heart and the thoracic aorta [14]. These can be assessed to detect past-myocardial infarction, dilated cardiomyopathies, atrial enlargement, aneurysms and pericardial effusion. However, coronary calcium scans contain a wealth of untapped information on other cardiovascular and non-cardiovascular health parameters [15, 16]. It is important for clinicians to be aware of the potential data on cardio-metabolic and general health that can be obtained from such scans without making any modifications to the scan protocol (Table 1). Hence, the goal of this review is to provide an overview of some of the most apparent imaging markers related to cardio-metabolic and general health. Additionally, we discuss potential incidental findings and radiation exposure of coronary calcium scans.

**Table 1:** Overview of imaging markers that can be derived from a coronary calcium scan

Cardio-metabolic health	General health
Coronary artery calcium (Agatston score, volume and density)	Vertebral bone density
Aortic valve calcification (Agatston score, volume and density)	Lung density
Mitral annular calcification (Agatston score, volume and density)	
Dimensions of heart chambers and ascending aorta	
Epicardial fat volume	
Liver density	
Pulmonary artery diameter	

## Markers of cardio-metabolic health

With the increasing focus on preventive medicine and the accompanying demand for individual risk stratification, the ability to calculate a patient’s risk of a clinical event relies greatly on the accuracy and amount of the acquired information. The coronary calcium scan can provide us with additional information regarding the patient’s cardiovascular health beyond the CACS. In the following paragraphs we address several of these markers.

### Coronary artery calcium volume and density

The Agatston-based CACS is a summary measure based on the total volume and density of epicardial coronary calcification into a single number ranging from 0 (i.e. the absence of calcifications) to scores of several thousand indicating extensive coronary atherosclerosis. However, more recent evidence suggests that calcium volume and density each separately harbour additional information with regard to the risk of subsequent clinical events [17–19]. Importantly, these measures of density and volume generally do not require additional processing or calculation, as these can be provided by most commercially available CACS scoring software. Moreover, the number and the regional distribution of calcifications can easily be visually assessed and provide additive predictive information regarding the future risk of major coronary events [20]. As a consequence, very recently a change in methodology to assess coronary calcium scans was proposed in order to incorporate this additional information into a new CACS [21].

### Valvular calcification

**Fig. 1:**
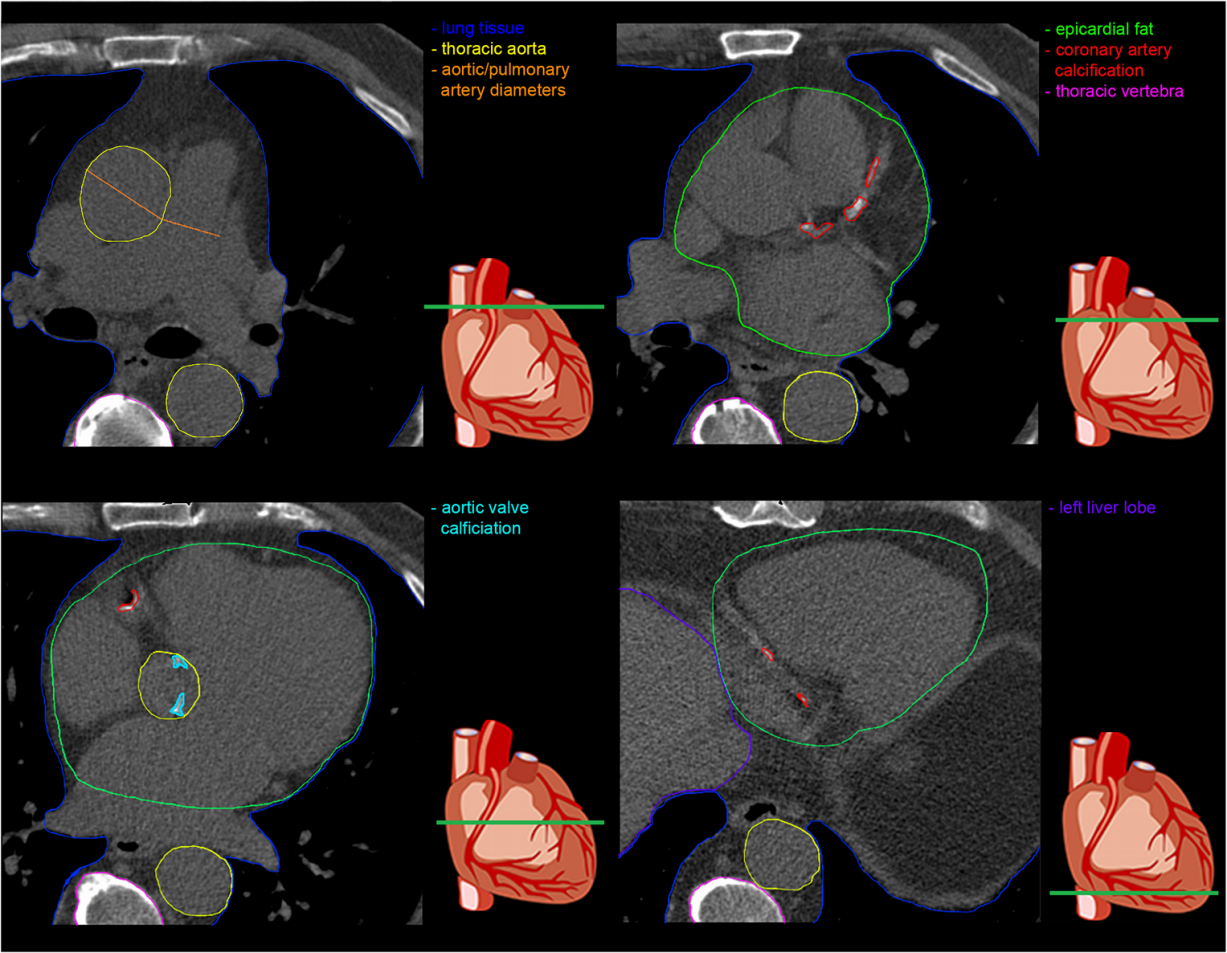
Imaging markers on non-contrast coronary calcium scans. Four slices of a coronary calcium scan of a single patient showing the heart at different levels with, in colour, the different tissues from which the potential imaging markers may be obtained

Using the same software as is used to obtain the CACS, one can quantitatively assess the burden of aortic valve calcification (Fig. 1, blue) [22, 23] or mitral annular calcification in the form of Agatston scores or volumes. The extent of aortic valvular calcification is a direct representation of degenerative aortic valve stenosis [24] and is associated with adverse cardiovascular outcomes and mortality [25, 26]. More specifically, recent evidence even highlighted that the load of aortic valve calcification measured by CT provides incremental prognostic value to predict aortic valve stenosis progression and subsequent occurrence of clinical events [27]. Similarly, mitral annular calcification, although less prevalent [28], was found to be associated with CACS [29], and to increase the risk of atrial fibrillation [30]. Additionally, progression of mitral annular calcification are an important predictor underlying left atrial abnormalities that predispose to atrial fibrillation [31].

### Epicardial fat

Epicardial fat is defined as the layer of metabolically active adipose tissue that surrounds the myocardium and the coronary arteries [32, 33]. Given this close anatomical connection, changes in the amount of epicardial fat may directly influence these structures. Larger amounts of epicardial fat are associated with more extensive coronary atherosclerosis [34–36], but also with direct arrhythmogenic effects on the myocardium in the form of an increased risk of new-onset atrial fibrillation and greater burden of atrial fibrillation [37, 38]. Due to rapid improvements in image-processing techniques it has become possible to quantify the amount of epicardial fat on non-contrast CT scans [39, 40]. These quantification methods are robust and fully automatic, but have not yet reached the same level of usability as commercially available software packages for calcium scoring. However, given the recent insights in the clinical importance of epicardial fat, implementation of tools for epicardial fat quantification in such software packages are expected.

### Liver density

In most instances, a coronary calcium scan also includes visualization of the upper part of the liver. Despite this being only a limited part of the whole liver, measurement of the mean attenuation value at two or three locations – which can readily be done using any CT-image viewer – appears to reflect the total amount of fat in the liver [41, 42]. In turn, the amount of liver fat is regarded as an important precursor of the metabolic syndrome, and is related to both subclinical and clinical cardiovascular disease [43, 44]. Liver density may also reflect subclinical hepatic fluid congestion and liver fat is associated with adverse cardiac remodelling, both of which may herald future heart failure [45].

### Pulmonary artery diameter

The diameter of the pulmonary artery (Fig. 1, orange) can be measured on non-contrast scans using any CT-image viewer and may be considered as a marker of pulmonary arterial pressure [46]. When adjusted for body size by comparison to the aortic diameter in the same slice (i.e. the pulmonary-artery-to-aorta ratio), increased pulmonary artery diameters are related to risk of future adverse pulmonary events and mortality, particularly in individuals with chronic obstructive pulmonary disease [46, 47].

## Beyond markers of cardio-metabolic health

In addition to aforementioned markers of cardio-metabolic health, other structures that are imaged provide additional information on for example fracture risk and the presence or risk of pulmonary events (Table 1).

### Bone density

With regard to measuring the bone density (Fig.1, pink), it should be acknowledged that apart from the heart, there may be considerable variation in the imaged area, depending on patient size and position. Yet, the majority of scans will include multiple thoracic vertebrae that can be assessed for bone mineral density – a key modifiable risk factor for osteoporotic fractures [48, 49] – or the presence of vertebral osteoporotic fractures [49].

### Lung density

Measuring lung density (Fig. 1, dark blue) as a direct marker of emphysema can be challenging, because in most clinical settings the field-of-view is narrowly set to visualize coronary calcium only [50]. Nonetheless, the overall lung density can generally be measured in the lower lobes of the lungs and in the areas surrounding the hila. However, it is important to mention that apart from this dedicated, narrow field-of-view, one may consider additionally reconstructing the coronary calcium scan with a wider field-of-view to also visualize all the lung tissue that was originally in the scan field. Although the tops of the lungs will still be missing, one can obtain an accurate impression of the status of the remaining part of the lungs with respect to the amount of emphysema [51, 52]. A downside of this wider field-of-view is the greater probability of detecting incidental findings.

## Incidental findings

When performing imaging, both in the clinical setting as well as in the research setting, incidental findings can be expected. However, the spectrum of potential incidental findings is relatively limited for coronary calcium scans [53]. Apart from cardiovascular abnormalities, incidental findings may especially be detected in the liver and the lungs. Given that no contrast is administered during a coronary calcium scan, potential findings in the liver are largely restricted to cystic lesions. However, for the lungs a substantial number of pulmonary nodules may be expected. Especially for older individuals and smokers, clear-cut criteria on the diagnostic work-up of such pulmonary nodules have been established and refined in the past decade [54, 55]. Other less frequent incidental findings may include interstitial changes of the lung, pleural effusion, chest wall abnormalities, breast calcifications and mediastinal lymphadenopathy.

## Radiation

A topic of concern accompanying the use of the coronary calcium scan is the ionizing radiation exposure to the patient or, in the research setting, to the study participant [56]. Two general key principles that should always be kept in mind when ordering a CT examination of any kind are justification in ordering the examination and optimization of the scan protocol in the way that the radiation exposure is as-low-as-reasonably-achievable (ALARA). With the newer generation CT scanners and improvements in scan protocols, radiation doses have been decreasing over the last few years and are expected to decrease further with advances in technology [57]. Specifically for prospective ECG-gated non-contrast coronary calcium scans, radiation exposure approximates 1.5 mSv (estimated using ImpactDose version 2.3, 2016, CT Imaging GmbH, Erlangen, Germany) [58, 59]. For comparison, the annual background radiation varies between 2 and 5 mSv. Nonetheless, radiation exposure should always be weighed against the information obtained from a coronary calcium scan. Following the ALARA principle in minimizing radiation exposure, it seems only reasonable to also force clinicians and researchers to transpose this principle to data acquisition once a scan is made: acquire as much as reasonably achievable relevant information from every imaging study.

## Conclusion

The clinical value of the CACS in terms of individual risk assessment of future cardiac events has led to an increased use of non-contrast cardiac CT in both clinical and research settings during the past decades. Many other markers of cardio-metabolic health and general health may readily be evaluated on these examinations. Clinical cardiologists, cardiovascular radiologists and medical specialists in the field of preventive medicine should be aware of this potential diagnostic and prognostic extra-coronary yield of the coronary calcium scan, and widen their professional field-of-view to look beyond the heart.

## Abbreviations

ALARA: As-low-as-reasonably-achievable
CACS: Coronary artery calcium score
CT: Computed tomography
ECG: Electrocardiography
